# Supplementary material for: Zinc ion flux during mammalian sperm capacitation
Source: Nat Commun. 2018 May 25;9:2061. doi: 10.1038/s41467-018-04523-y (PMC5970269; doi:10.1038/s41467-018-04523-y)
Supplement: Supplementary file 1 — Supplementary Information [file 41467_2018_4523_MOESM1_ESM.pdf]

Supplementary Information

Gating approach

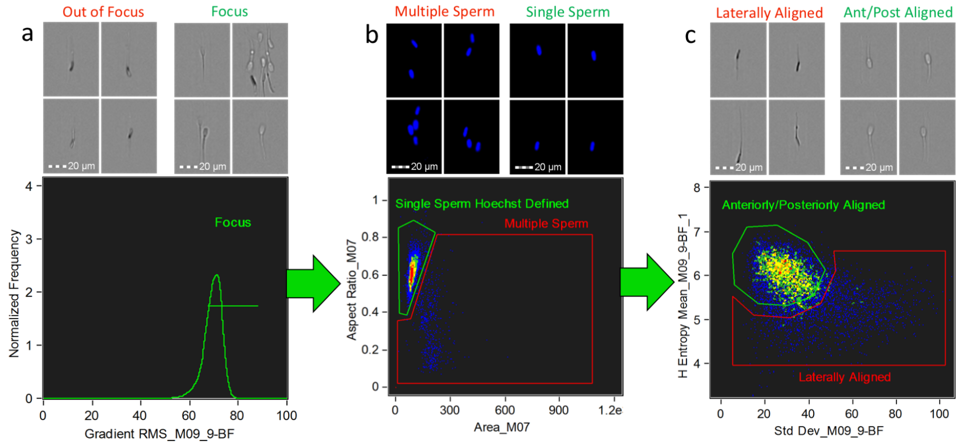

Masking approach

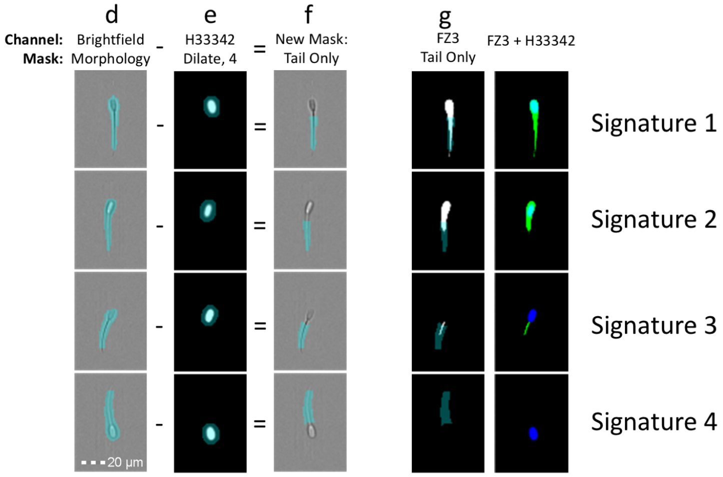

Gating and masking approach combined

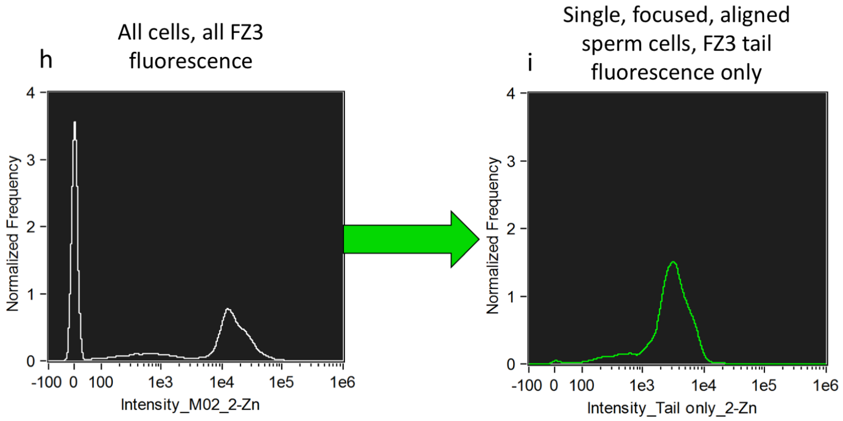

Supplementary Figure 1: Gating and masking approach.
a, Initial events collected from FlowSight data acquisition were gated for cells in focus as a function of brightfield gradient RMS, (a calculation of image crispness). **b**, Events in focus are further gated to analyze only single spermatozoa, plotted as H33342 fluorescence area by aspect ratio. **c**, To differentiate and gate out laterally aligned spermatozoa, a plot of brightfield standard deviation by brightfield H entropy mean is used. **d-g**, Mask (cyan) defines the region analyzed; fluorescence color removed to create greater contrast. **d**, Morphology mask of the brightfield **e**, subtracting a 4 pixel dilation of H33342 **f**, resulting in mask analyzing FZ3 fluorescence in the sperm tail only. **g**, Resulting gating and masking strategy ensures prudent data analysis not possible with conventional flow cytometry (all scale bars: 20 µm).

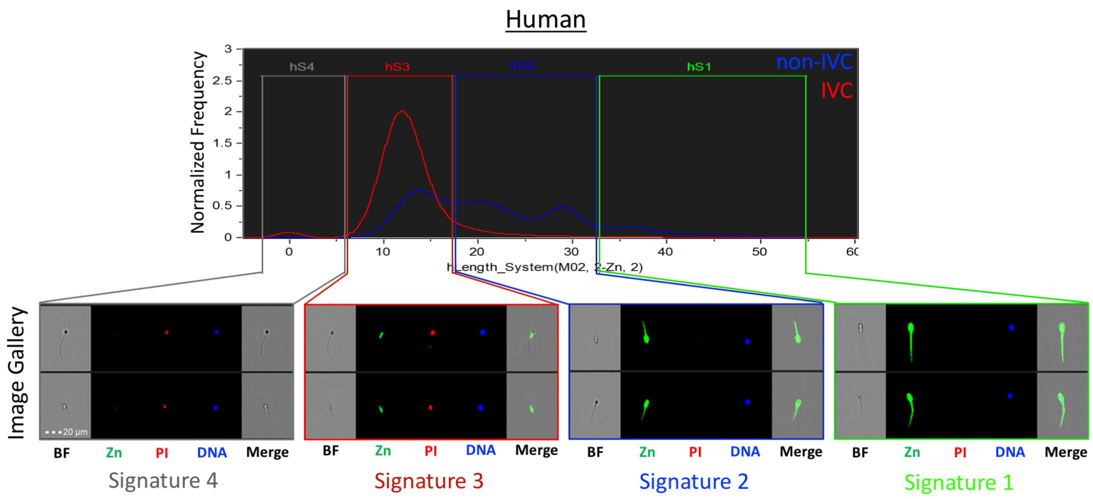

Supplementary Figure 2: Zinc signature of human spermatozoa after IVC.
Human spermatozoa undergo shift similar to porcine spermatozoa, towards signature 3 after 4 hours of IVC (histogram, red) compared to neat, non-IVC spermatozoa (histogram blue; scale bar: 20 µm).

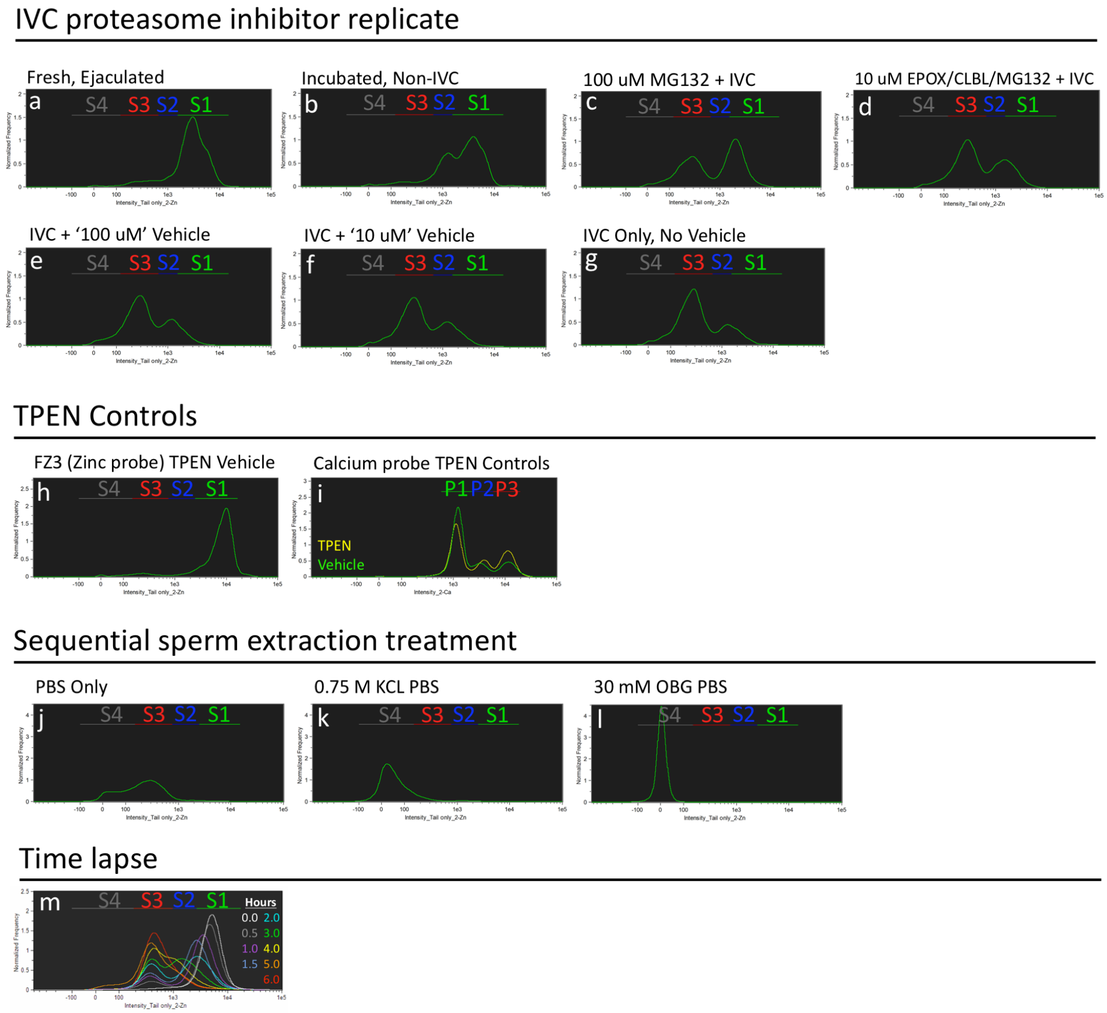

Supplementary Figure 3: Replicate, controls, and sequential extraction analysis. a-g, Second biological replicate of IVC proteasomal inhibitor treatments. **h**, TPEN FZ3 zinc probe vehicle treatment; vehicle did not shift zinc signature compared to no vehicle or TPEN treatment. **i,** Fluo4 calcium probe TPEN and vehicle control to show no decrease in TPEN-treated calcium levels. **j-l**, Sequential extraction of fresh, ejaculated spermatozoa. **j**, Most Zn-induced FZ3 fluorescence was lost in the first step (PBS wash), suggesting the bulk of Zn ions is freely available and can be depleted by a simple diffusion into the treatment buffer, observed by shift to signature 3. **k**, Increasing the ionic strength with 0.75 M KCl treatment resulted in further depletion of Zn^{2+}, after which the zinc signal was observed solely in sperm tail midpiece, where Zn metalloproteinases/Zn-dependent structural proteins are present within mitochondria, and Zn^{2+} is thus held by electrostatic forces. This agrees with further shift of sperm from signature 3 to signature 4. **l,** Introduction of non-ionic detergent (30 mM OBG) in the treatment buffer in the final step resulted in release of all remaining Zn, observed as complete shift of remaining signature 3 spermatozoa to signature 4 (complete loss of Zn-induced fluorescence). **m**, Time lapse Zn Signature cytometry every 30 minutes between 0-2 hours, then every hour until 6 hours; color code of time points located in figure legend.

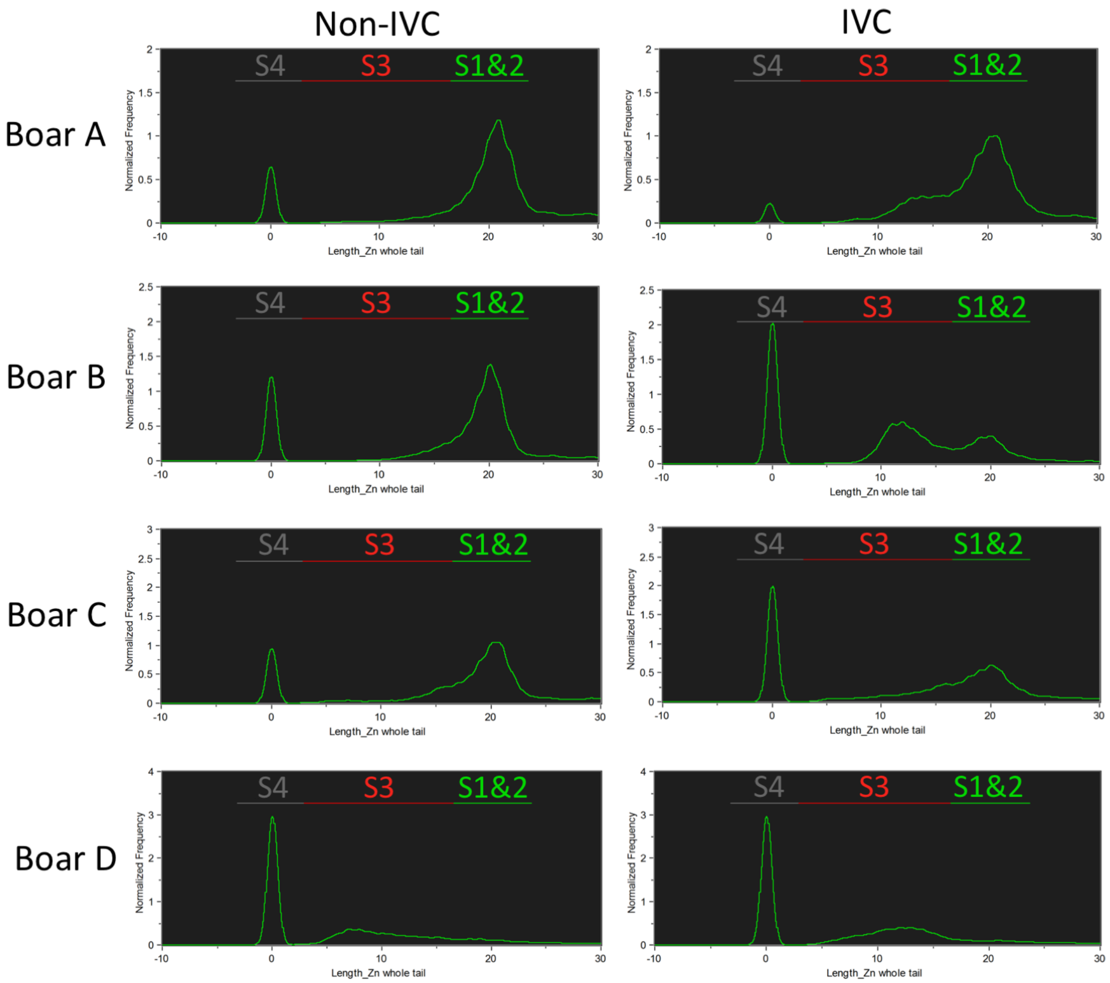

Supplementary Figure 4: Zinc signature fertility trial histograms.
a, Zinc signature histograms of four boars with known high or low fertility, before and after IVC.

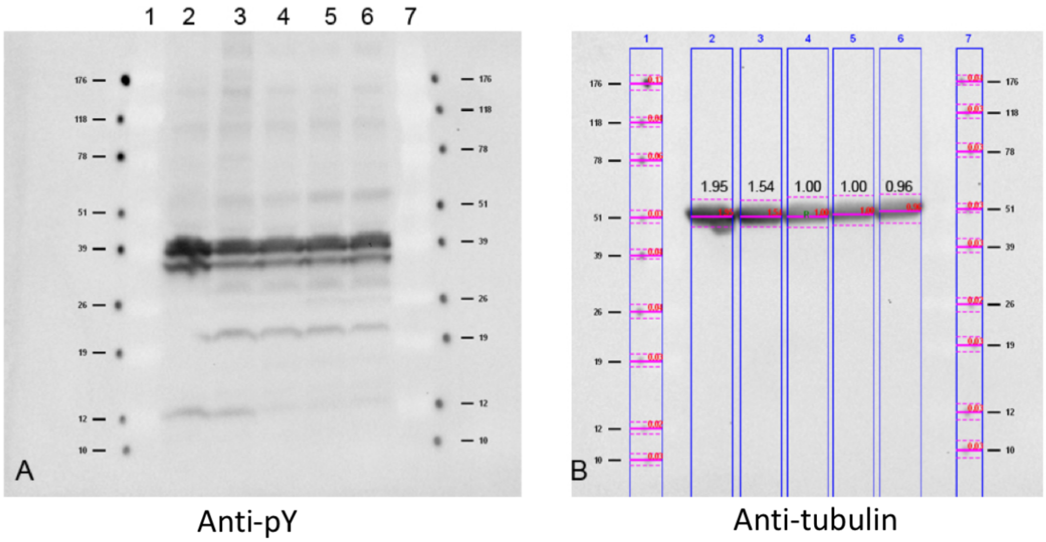

Supplementary Figure 5: Capacitation status confirmation under varied IVC conditions. The density of band migrating between 19-26 kDa markers (panel a) remained unchanged relative to loading control (panel b) in the extracts of spermatozoa capacitated with high vs. low sodium bicarbonate and pyruvate. **a,** Anti-phosphotyrosine Western blot of sperm extracts from treatments in the following lanes: 1) marker; 2) ejaculated, non-IVC; 3) experimental IVC conditions (2 mM sodium bicarbonate, 5 mM pyruvate); 4) comparison IVC (15 mM sodium bicarbonate, 0.2 mM pyruvate); 5) comparison IVC (15 mM sodium bicarbonate, 5 mM pyruvate). **b,** Loading control anti-tubulin Western blot with quantification for normalization.

Supplementary Table 1: Boar fertility trial records.

Boar	Farrowing Rate	Average Litter Size	Services (n)
Boar A	90.5%	10.03	42
Boar B	89.5%	9.52	38
Boar C	65.2%	7.40	23
Boar D	57.1%	7.17	21
