# Supplementary material for: Zinc ion flux during mammalian sperm capacitation
Source: Nat Commun. 2018 May 25;9:2061. doi: 10.1038/s41467-018-04523-y (PMC5970269; doi:10.1038/s41467-018-04523-y)
Supplement: Supplementary file 3 — Description of Additional Supplementary Files [file 41467_2018_4523_MOESM3_ESM.docx]

**Description of Additional Supplementary Files**

File Name: Supplementary Movie 1

Description: **Zona-bound motile spermatozoa have Zn signature 2.** On left, still image fluorescence of zinc probe FZ3 (green) and sperm head (blue) with MII oocyte acquired at the start of video recording (2.0 seconds acquisition time). Time lapse video recording of Zn-induced fluorescence was not possible due long acquisition time and rapid photobleaching. On right, differential interference contrast (DIC) video of the zona-bound, hyperactivated, Zn signature 2 spermatozoon at seven o’clock position (DIC final video output 30 frames per second).

File Name: Supplementary Movie 2

Description: **Hyperactivated spermatozoa have Zn signature 2.** Video imaging of sperm Zn signature state 30-60 minutes into IVC. On left, still image of fluorescence of zinc probe FZ3 (green; 2.0 seconds acquisition time) with video of spermatozoa DIC to right (DIC final video output 30 frames per second). Signature 1 spermatozoon displays normal, non-capacitating motility; signature 2 spermatozoon displays capacitation-induced, hyperactivated motility; signature 3 spermatozoon, presumed to be post-capacitated, shows no motility.

File Name: Supplementary Movie 3

Description: **Zn signature in motile vs. immotile spermatozoa after IVC.** Hyperactivated signature 2 spermatozoon shows both the head and the tail midpiece labeling, the latter having a fanned appearance due to hyperactivated tail movement and long acquisition time required to record the fluorescent signal. Post-capacitated immotile signature 3 spermatozoon has labeling restricted to midpiece. FZ3 acquisition for 2.0 second and DIC final video output 30 frames per second.

File Name: Supplementary Movie 4

Description: **Comparison of sperm motility under varied IVC conditions.** Left panel shows regular (as in not hyperactivated) sperm motility of non-IVC spermatozoa. Middle panel shows hyperactivated spermatozoa at 30 minutes after incubation under experimental IVC conditions. Right panel shows similar hyperactivated motility at 30 minutes after incubation in IVC medium containing 15 mM sodium bicarbonate. Final DIC video output 30 frames per second.
